# An Efficient Framework for Development of Task-Oriented Dialog Systems in a Smart Home Environment

**DOI:** 10.3390/s18051581

**Published:** 2018-05-16

**Authors:** Youngmin Park, Sangwoo Kang, Jungyun Seo

**Affiliations:** 1 Department of Engineering, Computer Science, Sogang University, Seoul 04107, Korea; pymnlp@gmail.com (Y.P.); seojy@sogang.ac.kr (J.S.); 2 Department of Software, Gachon University, Gyeonggi-do 13120, Korea

**Keywords:** dialog framework, smart home, intelligent virtual assistant, natural language understanding

## Abstract

In recent times, with the increasing interest in conversational agents for smart homes, task-oriented dialog systems are being actively researched. However, most of these studies are focused on the individual modules of such a system, and there is an evident lack of research on a dialog framework that can integrate and manage the entire dialog system. Therefore, in this study, we propose a framework that enables the user to effectively develop an intelligent dialog system. The proposed framework ontologically expresses the knowledge required for the task-oriented dialog system’s process and can build a dialog system by editing the dialog knowledge. In addition, the framework provides a module router that can indirectly run externally developed modules. Further, it enables a more intelligent conversation by providing a hierarchical argument structure (HAS) to manage the various argument representations included in natural language sentences. To verify the practicality of the framework, an experiment was conducted in which developers without any previous experience in developing a dialog system developed task-oriented dialog systems using the proposed framework. The experimental results show that even beginner dialog system developers can develop a high-level task-oriented dialog system.

## 1. Introduction

A smart home system is a system that monitors and controls various electronic devices to provide convenience to users in a private residential environment. For instance, a smart home system monitors and controls the climate in a residential setting such as temperature, humidity, and illumination in real time using various sensor devices, thus providing convenience to the user [1,2]. In recent times, technology has proliferated into various aspects of daily life. The widespread use of smart phones, tablet PCs, and high-speed internet, has led to active research on a spoken dialog system, which acts as an interface between users and a smart home system. The integration of a spoken dialog system with smart homes can provide a more convenient experience to the user. In particular, if the spoken dialog system can recognize user behaviors based on input from motion sensors, the dialog system can realize active dialog. For example, to automatically set the temperature and illumination in a house just prior to the user’s return to the house, the system automatically recognizes the user using sensors when he/she leaves home and initiates a conversation like “What time will you come home?” If the user replies, “I will come home at about seven p.m.”, the system can respond “Alright, I will turn the heater on at six thirty in the evening.” Thus, based on the response of the user, the system can customize the conditions in the house that are suited to the user’s preferences.

In particular, a task-oriented dialog system recognizes a user intended task from a user’s speech in a smart home environment and provides core functions to accomplish the intended purpose. Such a dialog system is designed to achieve specific objectives and tasks such as climate control, schedule management, hotel reservation, and text-based communication [3,4,5,6,7]. A task-oriented dialog system comprises the following five components: automatic speech recognition (ASR), natural language understanding (NLU), dialog management (DM), natural language generation (NLG), and text-to-speech synthesis (TTS). Each component must be configured to achieve the objectives of the dialog system. Depending on the services to be provided, the dialog system may require integration with other functional modules, because certain applications, in addition to the basic modules, require other functional modules to successfully perform operations. Without expertise in dialog models and natural language processing, it is rather challenging to effectively design such tasks, as developers will be required to implement each module separately for accomplishing different operations.

Most previous studies on task-oriented dialog systems have discussed specific modules in the proposed systems [3]. Although these individual studies have contributed to the improvement in the quality of such dialog systems, they cannot be directly applied to other dialog systems [8]. However, to address some of these issues, researchers are actively studying end-to-end dialog systems using deep neural networks [9,10,11]. Nevertheless, this approach cannot be directly used for developing a practical task-oriented dialog system because of scalability issues. Thus, there is a need for a dialog framework that integrates and manages each component of the system to develop a task-oriented dialog system. Unfortunately, relevant studies on this topic are scarce.

Considering this, in this study, we propose a dialog framework that helps non-expert developers in dialog systems and natural language processing implement a task-oriented dialog system. The proposed dialog framework provides the following differentiated functions:

- A rule-based approach to build the basic components of the dialog system (SLU, DM, and NLG), and expressing the rules used in the dialog knowledge. The dialog knowledge can be easily understood because it is structured in an ontological style, and basic conversational agents can be implemented by editing it.
- A module router that can link the system with external modules to add various functions without accessing the source code of the dialog system. The module router is executed by calling external modules based on the conditions set by the developers.
- A hierarchical argument structure (HAS) to manage various argument representations of natural language sentences.

The remainder of this paper is structured as follows: Section 2 reviews the research related to task-oriented dialog systems. The structure and features of our proposed dialog framework are described in Section 3, while Section 4 introduces the dialog models and dialog knowledge that are the core components of our research. Section 5 explains the NLU and NLG methods used in the proposed dialog system. Section 6 describes the experimental procedures and results, which evaluate the usability of the proposed dialog framework. Lastly, Section 7 presents the conclusions and directions for future research.

## 2. Related Work

**Figure 1:**
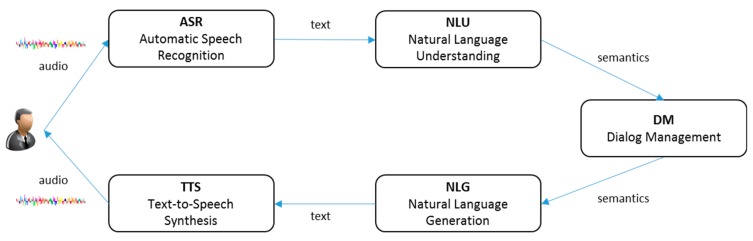
Overview of task-oriented dialog system.

This section discusses previous work on the components of a dialog system as well as on frameworks for building an effective dialog system. Figure 1 shows the typical structure of a task-oriented dialog system. Owing to the comprehensive and cumbersome nature of covering every component of this structure, most studies have addressed individual components. Previous research primarily focused on improving the performance of the system by applying machine learning(ML) models such as the Bayes classifier [12,13], support vector machine (SVM) [14], maximum entropy (ME) [15], and conditional random fields (CRF) [16]; some studies have also used feature engineering. However, recently, the application of deep neural network (DNN) to improve the performance of such task-oriented dialog systems has gained momentum [17].

Studies related to domain/intent detection primarily involved the application of machine learning-based classification models such as SVM and ME [18,19]. More recently, the application of DNN models has also gained interest. In [20], the authors describe the use of mass unlabeled data belief in an initialization task for a deep belief network; the results showed improved accuracy when compared against existing classification models. Since then, the application of recurrent neural network (RNN) models and convolutional neural network models to domain/intent detection has been regarded as the state-of-the-art [21,22]. Further, research related to slot-value detection primarily involved performing sequence labeling that attached begin, inner, and outer type tags to the corresponding word positions. These studies assumed that values such as date, time, and location, which are required to perform domain/intent detection, are present as it is in a given sentence. In [23], the authors proposed a slot-value detection method using conditional random fields, which showed excellent performance in sequence labeling; in addition, they introduced a feature engineering method to improve the performance of slot-value detection accuracy. Then, slot-value detection using long-short term memory (LSTM) was proposed [24]. In recent studies, sequence-to-sequence models have been widely applied for machine translation [25,26]. In addition to these studies, studies have been conducted that combine domain/intent and slot-value detection methods to reduce the propagation of errors [27] and effectively address the ambiguity using contexts, which reflect dialog history information [28].

The role of DM involves dialog state tracking (DST), which updates the dialog state when a conversation is in progress, and a dialog policy (DP), which creates a dialog act to respond to the user in the corresponding dialog state. Rule-based DM is a way of manually defining the possible states of a dialog system and the behavior of the system for the given state. Finite state (FS)-based DM [29,30], one of the typical rule-based DM, is the simplest and widely-used method to date. FS-based DM component converts the finite state automata (FSA) based on the output of the NLU, and generates dialog act based on this output; such is achieved by manually designing an FSA to analyze a conversation scenario. Meanwhile, information state (IS)-based DM [31] provides more generalized and flexible functions compared to FS-based DM. IS contains various dialog context sets (such as task, slot-value, dialog history, ASR confidence score etc.) and update rules. Then IS-based DM selects the update rule matched with context set, and updates the IS during conversation. Rule-based DM is significantly intuitive; its advantages include easy addition of new domains and easy maintenance. However, rule-based DM is vulnerable to errors in ASR and NLU, because it is entirely dependent on the result of the NLU to recognize the intent of the user. Therefore, it is important to robustly complete a conversation analysis even for semantically ambiguous input. Therefore, to address this issue, active research is being conducted to track the state in such FSA while maintaining the dialog state using a probability distribution. In particular, Young [32] showed that an FS-based DM component could be robust against errors in ASR and NLU by successfully applying a partially observable Markov decision process (POMDP) to the DM. Annually, the dialog state tracking challenge (DSTC) [33] is held, where researchers compete against each other based on the performance of their proposed systems on selected new issues related to DST. In [34], Henderson improved DST accuracy by building a multi-layer perceptron and applying rich feature representations. Another study [35] used both NLU and ASR results as features to supplement the information lost in the NLU process. Moreover, methods using reinforcement learning (RL) have been primarily proposed for DP optimization [36]. In such methods, a reward must be assigned to the output from the model. For this, either a user simulator that plays the role of a user is developed or a method of seeking an actual user for the reward is used [37]. In [38], a POMDP-based dialog manager was proposed that simultaneously applied rule-based dialog management and probabilistic dialog management.

The most widely used method in natural language generation (NLG) is a template-based NLG. The template-based NLG is a method for defining the sentences that can be output for all the possible output dialog acts in DM. For this, appropriate rules that allow certain changes based on the values of dialog acts are applied to the sentences [39]. The main advantages of the template-based NLG method include the low number of errors and the ease with which developers can control the system. However, building such systems is cumbersome when there are several dialog act types; this can be regarded as a drawback. Moreover, it cannot process undefined dialog acts. To address these problems, recently, a machine learning-based NLG has been actively researched. In [40], Mairesse proposed a method to perform NLG using a Bayesian network. This study defined a new tag, called a semantic stack, by dividing a natural language sentence into phrase units, and created a sentence using the Bayesian network. In addition, it was shown that their method could achieve higher performance even when annotating small data using active learning. However, recent research has primarily focused on using a corpus consisting of only dialog acts and sentences because tagging the semantic stack is a relatively expensive annotation task. In [41], Wen proposed a method to generate a sentence using the one-hot encoded representation of the dialog act as the initial hidden state of the RNN in a manner similar to image captioning [42]. A subsequent study [43] showed improved performance by proposing a semantic conditioned LSTM in order to prevent the repetition of words with the same meaning or including information that specific dialog act values are not present in sentences. Then, in [44], Press showed that the generative adversarial network (GAN) that was used quite effectively in image recognition could also be used in NLG.

Studies on the dialog framework that helps the development of the task-oriented dialog system have not been widely conducted because the studies are considerably difficult. Nevertheless, studies on an effective dialog framework have been steadily underway. Of these, VoiceXML [45], RavenClaw [46], and DialogStudio [47,48] are the frameworks that have been used to develop actual commercial dialog systems. VoiceXML is a markup language that can define and express the dialog flow specified by the World Wide Web consortium (W3C), thus dialog management can be implemented based on VoiceXML. In [49], implemented a dialog system based on VoiceXML and Galaxy HUB. However, VoiceXML is very inflexible and implementing a relatively complex dialog system is challenging. RavenClaw primarily involves plan-based dialog management, and yields the knowledge output, known as a dialog task tree, which indicates the purpose of a dialog; in this case, a dialog engine plays the role of planning the tasks to be executed based on the content of the dialog task tree when interacting with the user. In addition, it has been proven through various implementation cases that this method can be used in various commercialization services. However, to build a dialog system based on RavenClaw, it is necessary to manage relatively complex knowledge grammar and build many rules. DialogStudio is focused on providing a corpus-based dialog system using an example-based dialog management method; in other words, if we have a sufficiently large dialog corpus, we can implement a dialog system without building complex knowledge. However, the example-based dialog management method has some drawbacks in that it is difficult to accurately control the dialog system and guarantee the performance when the corpus is not sufficiently large. Until recently, framework studies helping implement dialog systems have been underway. Owlspeak [50] is an ontology-based dialog framework that uses Web Ontology Language (OWL) to generate VoiceXML documents dynamically. Owlspeak provides a model-view-presenter (MVP) design pattern: its model represents dialog knowledge, and its view updates dialog state and generates the dialog act of the following system. Then the dialog act of the system is rendered in the view to Voice XML document. The framework proposed in [51] focused on helping quickly implement machine learning-based dialog management and natural language understanding, and provided a special function called a story graph that visualized the flow of dialogue scenarios in advance. In [52], the authors proposed a framework for expressing dialogue behaviors as probabilistic rules. The probabilistic rules used in this study consist of conditional statements and actions with probability; these probabilistic rules can be made manually or generated automatically by supervised learning or reinforcement learning. In [53], a very high quality framework is proposed that provides all the necessary tools, such as natural language understanding, dialog management, language generation, evaluation, user simulation, etc.

## 3. Dialog Framework for Task-Oriented Dialog System

This section describes the structure of the general task-oriented dialog system and the proposed dialog framework. Figure 1 shows an overview of the general task-oriented dialog system. ASR generates text by recognizing the user's voice signal. NLU component generates semantic tags by analyzing the grammatical structure and semantics of the text generated by ASR. The DM component consists of a dialog state tracker and dialog policy. The dialog state tracker updates the arguments of the dialog state to be executed using semantic tags. The dialog policy generates semantic tags to respond to the user in the current dialog state. The NLG component generates natural language sentences from semantic tags that the user can understand, while the TTS component generates speech signals from natural language sentences that the user can hear. Our proposed dialog framework covers NLU, DM, and NLG components, and excludes the ASR and TTS components in a general task-oriented dialog system.

### 3.1. Dialog State

**Figure 2:**
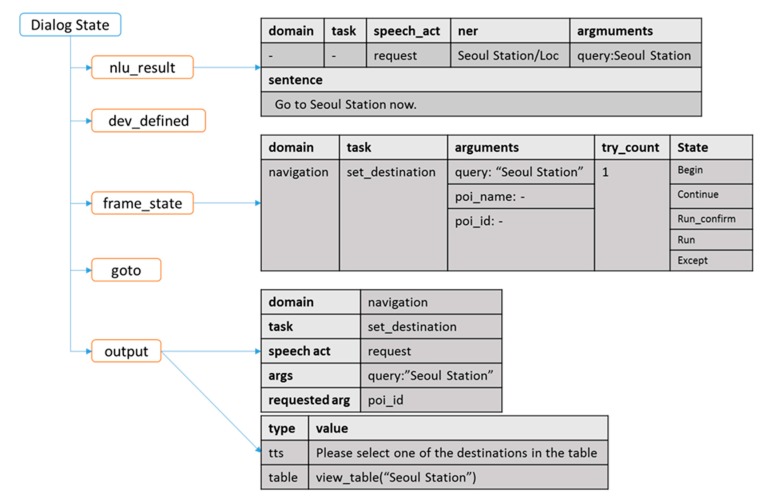
Dialog state properties.

All the modules that constitute a dialog system operate independently. However, input and output information must be synchronized because the modules must operate as a single system. In our proposed framework, this is achieved by the dialog state. All modules can reference and record only the information of the dialog state. Therefore, the conflict on routines with other modules is minimized even if the function of a specific module is added or modified because the structure allows all modules to be executed independently. Figure 2 shows the structure of the dialog state. The role of each field is as follows. The nlu_result field contains the result of the NLU analysis of the current input sentence; in general, it is updated by the NLU modules. The frame_state field contains the module information for ongoing conversations and is primarily updated by the DM component depending on the content of the nlu_result field. Further, the dev_defined field is a data structure with a map structure, which can be used by developers to record and reference values as necessary. The goto field redirects the dialog state to a specific step once the execution of the current module is completed; this redirection can also include the previous step. The dev_defined and goto fields play an important role in the module router, which will be described later. Lastly, the output field contains the response information, which comprises a system dialog act and a realized template for the user; it is generated primarily by the NLG component.

### 3.2. External Modules and Module Router

A commercial dialog system uses various functions as well as a basic dialog process. For example, in a conversation for GPS navigation, the user should be able to search for a destination during the conversation and perform various actions depending on the results. As another example, in a conversation for schedule registration, the user should be able to perform various actions depending on the values registered in the current calendar. All these functions are essential in a practical dialog system. To realize such functions, it is necessary to build a dialog system anew at the source code level; thus, developers can implement the dialog system effectively only if they have experience developing a system on their own or have a deep understanding of each module. As the number of added functions grows, the difficulty in managing these functions also increases. Thus, to address this complexity, in this study, external modules and a module router are proposed that enable developers to perform this task without accessing the source code of the basic dialog system. These functions allow the modules to be managed in a manner similar to the separation of the rules used in the process of transforming dialog from the dialog system into dialog knowledge, which, in consequence, is similar to the separation of the added source code from the dialog system.

**Figure 3:**
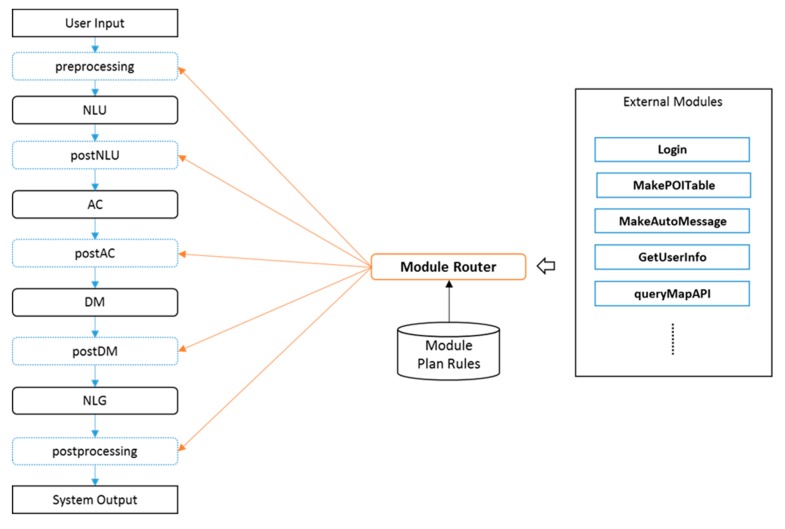
External modules and module router.

The dialog system described above consists of four steps: NLU, argument generation (AG), DM, and NLG. The proposed framework adds intermediate steps for developers to enable intervention between steps. Thus, the process step of the dialog system has the structure shown in Figure 3. Developers can implement external modules and execute them by creating module plan rules, if necessary. In this case, external modules cannot access the source code of the dialog system, yet they can control the operation of the dialog system by referencing or recording values while accessing the dialog state.

**Figure 4:**
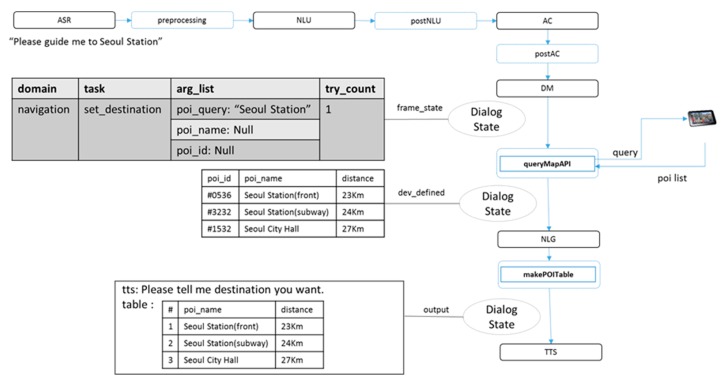
Example of external modules for navigation domain.

The conditions under which each external module is executed are defined in the module plan rules. The module router executes those external modules that meet the conditions of the dialog state by checking them at every step. Figure 4 shows an example of the executed external modules and module plan rules in a search for a destination in a GPS navigation application. If a user inputs the sentence “Please guide me to Seoul Station”, the module router will execute queryMapAPI in the postDM step based on the module plan rules. The queryMapAPI module performs a destination search using the poi_query argument using a map API and records the resulting information in the dev_defined field of the dialog state. Then, NLG generates the output list from the template, including the response of the table type. The module router executes MakePOITable in the post-processing step. The MakePOITable module uses dev_defined to generate a table. Finally, the user is provided with an output statement and a search result table. Thus, the proposed method allows external modules to access only the dialog state using the model router, thus enabling the addition of various functions through indirect coding.

## 4. Dialog Manager

A dialog manager consists of a dialog state tracker, which updates the contents of the frame state based on the result of the NLU analysis, an argument generator, and a dialog policy. The dialog policy generates dialog actions with which the system can respond to the user based on the current frame state. The dialog manager in the proposed framework uses FS–based DM [29], one of the rule-based DMs. With FS-based DM, it is easy for developers to intuitively create models, and add or modify for maintenance. Although some advanced rule based-DMs offer more advanced features, FS-based DM with module router can implement similar functionality to other rule-based DMs. Thus, the simplest FS-based DM is utilized to prioritize development convenience and robustness. For reinforcement learning or end-to-end models, it is necessary to collect a large quantity of data to create models. Furthermore, it is also difficult to verify usability of the dialog system. Because of the problems mentioned above, FS-based DM is actively applied in current dialog systems. With the FS-based DM component, the proposed framework can easily manage dialog system.

### 4.1. Dialog Knowledge

**Figure 5:**
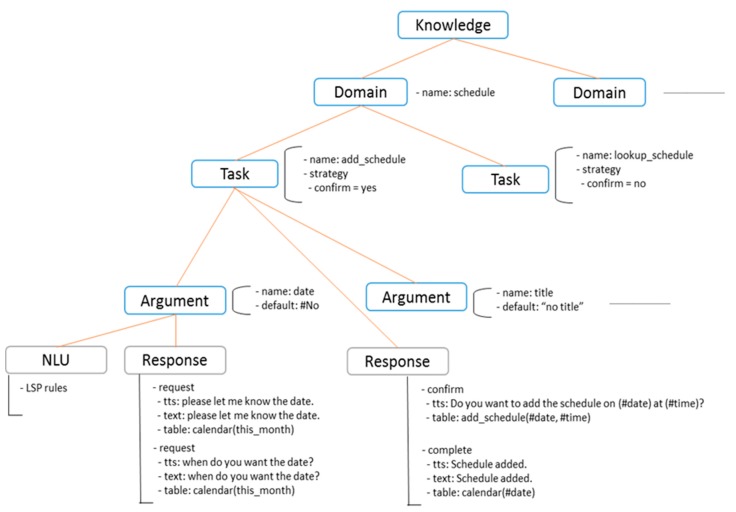
Structure of dialog knowledge.

In a task-oriented dialog system, dialog knowledge expresses information about the objective and strategy of the dialog system. Using this dialog knowledge expression, developers can construct the operational structure of the dialog system. Therefore, dialog knowledge should be easy for developers to understand and manage and should be as autonomous as possible. In general, dialog knowledge expresses information required to construct the DM component; however, in our proposed dialog framework, the rules used in NLU and NLG are included in the dialog knowledge. The proposed knowledge expression follows an ontology-based structure and can be managed independently according to the domain. An example of dialog knowledge is shown in Figure 5. The dialog knowledge consists of multiple domains, each of which consists of multiple tasks. Each task has multiple arguments, which are the information needed to perform the task.

### 4.2. Argument Generator

An argument that is the target of a task refers to some information needed to perform the task. The arguments of each task are obtained by the slot-value detection operation of the NLU component; in this case, the generated value is a word string present in a natural language sentence. However, this value cannot be used as-is in external applications. For example, if the result of the named-entity recognition (NER) analysis of the input sentence is “Wake me up at (four am)/time (tomorrow)/date”, the information required to perform an actual service should be a normalized value such as “time: 0400, date: 1108”. Therefore, a function is required to convert the NER result into a normalized argument value. The proposed framework provides an argument generation (AG) module that plays this role. AG uses a lexico-semantic pattern (LSP) [54] that is similar to the rule-based approach of NLU to perform this function.

### 4.3. Hierarchical Argument Structure

Among the natural language sentences present in the actual dialog system, the argument information is represented in various forms. In some cases, it does not include all necessary information but expresses some partial information. For example, in a sentence such as “please schedule for next week”, “next week” is date information, but only provides partial information. In this case, the same response requesting the date value is repeated, which may lead to the deterioration in the quality of the conversation because the argument value cannot be generated in the general argument structure. A more accurate response in this situation is to request the remaining partial information such as “Which day of next week do you want?” Therefore, in this study, we propose a HAS for the flexible operation of such argument expressions.

**Figure 6:**
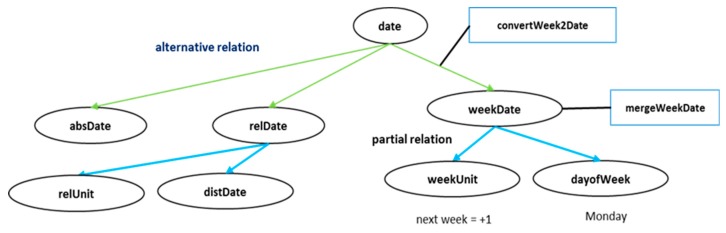
HAS example for date.

Figure 6 shows an example of the HAS for dates. Like the dialog knowledge, HAS also has an ontological structure. HAS uses two relations: an alternative relation and a partial relation. First, the alternative relation indicates that the parent argument can be represented by several alternative argument expressions. A date, for example, can be expressed as an absolute date, such as “December 28”, or as a relative date, such as “today” or “tomorrow”. Expressions such as “Monday of next week” are also possible. Thus, the date is connected to date.absDate (absolute date), date.relDate (relative date), and date.weekDate (day of the week) through the alternative relations. Second, the partial relation indicates that the parent argument can be represented by a combination of several partial arguments. For example, to express the date.weekDate “Monday of next week”, “next week” and “Monday” can be independently expressed, and date.weekDate can be completed when both partial expressions are present. Thus, it can be seen that the date.weekDate.weekUnit and date.weekDate.dayofWeek arguments are connected to each other through the partial relation to date.weekDate.

Using the proposed HAS, developers can design the task arguments more conveniently and provide more intelligent responses in such situations, that is, even if the user uses partial expressions. For example, when a user makes a request such as “please schedule for next week”, date.weekDate.weekUnit will have a value of “+1” after NLU and AG are performed. In this case, it can be seen that the request argument should be date.weekDate.dayofWeek based on the search result for the date argument. Therefore, the DM component will set the request argument of the response dialog action to date.weekDate.dayofWeek instead of date. A more intelligent conversation is possible by defining “Which day of (#date.weekDate.weekUnit) week do you want?” in the template of this dialog action.

The HAS was used to obtain both date.weekDate.weekUnit and date.weekDate.dayofWeek values from the user, but the final value applied as the date argument must be an absolute date value, such as “20180226”. For this task, the HAS executes the merge module for the partial relation and the conversion module for the alternative relation. First, the merge module generates a parent argument by combining the values of all arguments connected through the partial relations of a specific argument. In the case of date.weekDate, for example, mergeWeekDate will be executed, and date.weekDate will have the value “Monday, +1” from the partial relation values. When any of the arguments connected through the alternative relation of a specific argument has a value, the conversion module converts that value to the parent argument. For example, if date.weekDate has a value of “Monday, +1”, then convertWeek2Date is executed, and the converted date value is “20180226”. Consequently, the date argument now has an absolute date expression. Because the modules used in this process can be defined and connected to the ontological structure by developers, they can also be used for arguments other than date.

There are various arguments that can effectively apply HAS, for instance, in a smart home environment. For example, let us consider target_light in the turn_light task that controls the lights in the house. If there are several types of lights in the house, the room_type and light_type can be configured as partially related arguments of target_light. At this point, if the user requests “Turn on the mood light”, then the target_light.light_type is set to “mood light”, and it replies with a question like “In which room do you want me to turn on the mood light?” requesting the target_light.room_type. Thus, using the proposed HAS, we can build a more intelligent dialog system.

## 5. NLU and NLG

### 5.1. NLU with Hybrid Approach

**Figure 7:**
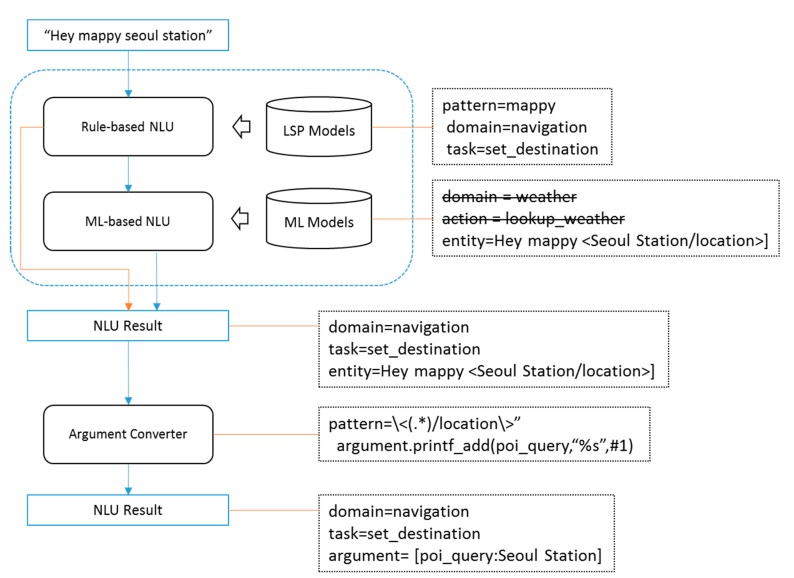
Example of NLU and AC.

In the proposed dialog framework, NLU consists of domain classification, task classification, speech act classification, and NER. Rule-based or machine learning approaches can be used for each module, however, each approach has advantages and disadvantages for. In general, a rule-based approach enables high scalability and intuitive comprehension because developers create rules manually. Therefore, high accuracy for a given sentence pattern can be achieved in a short time when new domains and tasks are added. In addition, it has a major advantage in terms of maintenance because the reason for the analysis result can be intuitively understood and an error can thus be easily corrected. The drawbacks of this approach are its high costs due to the manual creation of rules and its relatively low overall accuracy. In particular, the dialog system has a large number of domains and the larger the scale, the more rules the dialog system needs which makes it more difficult to manage them. In addition, each of the rules can negatively interfere with each other, which may degrade performance. Rule-based approach can also be vulnerable in spoken dialog environment, because it is difficult to respond to ASR errors or exceptional input. On the other hand, if the machine learning approach is used, automatically trained models can be created by collecting a sufficient quantity of labeled data. This approach has the advantage of providing a relatively high degree of accuracy and is more cost effective than creating rules manually. In particular, the machine learning approach has advantages of cost and performance compared to rule-based approach in large-scale dialog system. It can also respond relatively well to ASR errors or exceptional inputs. However, if the quantity of labeled data is insufficient, it is difficult to ensure high performance. Moreover, it is relatively difficult to find and correct the cause of an analysis error. The advantages and disadvantages of the two approaches in NLU are clear, and it is desirable that we gain the benefits of each approach. Therefore, the proposed dialog framework provides all the advantages of both approaches by simultaneously applying the rule-based and machine learning approaches. Rule based and machine learning approaches can be applied simultaneously in two ways. The first is to apply machine learning-based NLU and apply rule-based NLU when the confidence score of result of NLU module is over the threshold score. The second method is the first method in reverse. It is difficult to deal with a specific situation with the first method because it is difficult to estimate the results of machine learning-based NLU. On the other hand, the second method handles specific situations using rule-based NLU, and it manages general situations with machine learning-based NLU, which makes it easier to control scenarios. Thus, the proposed framework employed the second method. Each NLU module first outputs the results using the rule-based approach based on an LSP regular expression. Machine learning is performed for the modules that cannot output results from the analysis by the rule-based approach. The results of the NLU analysis are recoded to the nlu_result field of the dialog state after merging the results of the two approaches. Figure 7 illustrates an example of the NLU analysis process.

### 5.2. Template-Based NLG

The dialog action generated by the DM component consists of semantic tags for the dialog system, which are used for responding to the user. Therefore, the semantic tags must be converted into natural language sentences or expressions that the user can recognize. Although research on RNN-based NLG models has been actively conducted recently, these models are not yet suitable for practical use. In particular, NLG can drastically degrade the conversation quality in the cases of low performance. To address this issue, the proposed framework generates content for responding to the user using a template-based NLG model. However, such a template-based NLG model required developers to manually create a response template for each case that the system can respond to. However, it can ensure high performance and is easy to maintain because the developers can intuitively understand the operation of the dialog. The template can be built to output not only text but also a variety of other output types, such as tables and images.

## 6. Experiments

This section describes the experimental procedures and results for verifying the usability of the proposed dialog framework. Experiments were set up to develop a multi-domain task-oriented dialog system for the Korean smartphone. First, three developers who had never developed a dialog system were trained in the structure and use of the dialog framework for a total of 30 hours over the course of a month. The building of a dialog system for simple scenarios was included in the training. After training, approximately six conversation domains in a smartphone environment were selected: call, message, weather, navigation, TV_guide, and schedule. One to six tasks were defined for each domain. For ASR, we employed the Google Cloud Speech API (https://cloud.google.com/speech). A hybrid approach was applied to the NLU modules of the dialog system by implementing both the rule-based and machine learning approaches. For NLG, a template was created for each situation. The dialog system had been under development for approximately six months. The development period includes training on the framework, domain selection and dialog scenario design, data collection, external application implementation, external API integration, and performance testing. The six-month development period may not seem like a short time to prove the usefulness of the framework. However, considering that the subjects of experiments were not experts in the dialog system and given the quality of the dialogue system developed by them, the six-month development period does not seem significantly long.

In [55], readers can find a video that shows the functioning of the system with actual smartphone applications. The number of rules and templates and the number of dialogs used in the machine learning approach are listed in Table 1.

**Figure 8:**
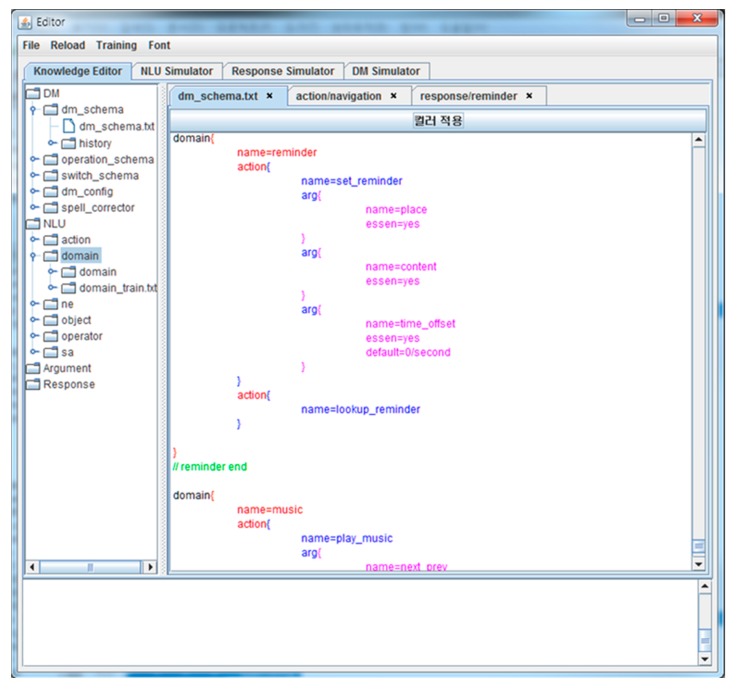
Screen shot of dialog knowledge editor.

The proposed framework was implemented in a JAVA 8 development environment. The framework provides a text-based editor for editing domains, tasks, arguments, NLU rules, NLG templates, etc., i.e., dialog knowledge. The editor screen is shown in Figure 8.

### 6.1. Evaluation

Quantitative and qualitative evaluations of the developed dialog system were performed. For quantitative evaluation, we used the accuracy of NLU modules. On the other hand, for qualitative evaluation, we used the success rate and user satisfaction as parameters.

#### 6.1.1. Accuracy of NLU Modules

As regards the quantitative evaluation, the accuracy of the NLU modules was measured. The models used in NLU were compared based on the approach used: the rule-based approach constructed by the developers, the machine learning approach [18] created using the training corpus, and the hybrid approach combining the two methods. Here, the machine learning approach can use other alternative classification models, thus varying the output accuracy of NLU. Eighty percent of the corpus was used for machine learning and for creating LSP rules to build each model; the remaining 20% was used for performance evaluation. The evaluation metric for domain, intent, and speech act detection was accuracy; that is, the percentage of correct predictions for each sentence. The evaluation metric for argument detection was the F1 score, which is the harmonic average of precision and recall. Precision, recall, and F1 score are defined in Equations (1)–(3), respectively:

(1) $$\mathrm{precision}=\frac{num\;of\;correct\;predictions}{num\;of\;system\;predictions}$$

(2) $$\mathrm{recall}=\;\frac{num\;of\;correct\;predictions}{num\;of\;gold\;arguments}$$

(3) $$F_{1}\;\mathrm{score}=2\cdot \frac{precision\cdot recall}{precision+recall}$$

The results are shown in Table 2. It can be confirmed that the overall accuracy decreased because the rule-based approach is analyzed only by the LSP built by the developers. The low accuracy may be attributed to the fact that the rule-based approach does not provide analysis results for patterns that are not predefined. Therefore, the overall recall is low, which decreases the performance of the F1-score. However, because of the nature of the rule-based approach, it can provide higher performance and several advantages, especially in terms of maintenance, if the LSP rules are accurately constructed. The overall accuracy was the highest with the machine learning approach. The hybrid approach was confirmed to provide performance similar to the machine learning approach despite a slightly lower relative performance. Although there are patterns that are not predefined, the low recall can be complemented because the ML model can handle such patterns. In addition, the precision is similar to the machine learning-based approach, thus showing similar overall performance. If the rules are more sophisticatedly constructed, the hybrid approach can outperform the machine learning-based approach. These findings indicate that the hybrid approach effectively provides all the advantages of the two models.

#### 6.1.2. Conversation Success Rate and Satisfaction

A qualitative evaluation was performed based on the conversation success rate and conversation satisfaction. Also in order to measure the effectiveness of the proposed HAS, the comparison was made with models with and without HAS. For the test, the HAS arguments were designed with location (for weather and navigation domain), date and time (for schedule domain). The conversation success rate refers to the rate at which the objective of a conversation is achieved when started with a specific purpose. In this study, a total of 100 conversations were attempted; 10 purposes were assigned to 10 different users. Here, the maximum number of conversation turn for each conversation is specified as the number of task arguments. If it exceeds the maximum number of conversation turn, it is regarded as a failure. Then, the evaluation metrics, success rate, is defined as shown in Equation (4).

(4) $$\mathrm{success}\;\mathrm{rate}=\frac{num\;of\;success\;conversaiton\;}{num\;of\;tried\;conversation}$$

The results listed in Table 3 demonstrate a conversation success rate of approximately 90% for without HAS and 91% with HAS. However, it is difficult to measure the objective performance, because the conversation success rate depends strongly on the performance of ASR and NLU, and thus, the accuracy tends to increase as the user gains more experience.

Conversation satisfaction was measured based on scores given by users after the functions of the developed dialog system were described to them and they were allowed to use the system freely. Ten users participated in the evaluation and performed 10–30 tasks. To evaluate the usability, the users assigned a score from 1 to 5. Table 3 indicates that the score for each domain was approximately 3.4 and 3.6. These results suggest that even though it was built by developers with little experience in the dialog model, the developed dialog system provided high-quality performance. In addition, it can be observed in the qualitative evaluation that the conversation success rate is not highly related to the satisfaction level. Although the conversation success rate is low, it resulted in relatively high satisfaction levels for the domains that are mostly likely to help people more profoundly in real life.

Comparing the case with and without HAS, model with HAS demonstrates some performance improvement in measuring success rate, because testers have succeeded in conversation by using different argument styles. More specifically, if model without HAS does not recognize a partial date representation (such as “next week”), the system will respond with a date request. At that time, the user can try the partial expression once more, or try another expression (such as “Monday, next week”, “May fourteen”) that the model can recognize. As a result, the improvement of success rate was not significant because the latter case was more frequent than expected. On the other hand, in measuring satisfaction, model with HAS demonstrates significant improvement, and is especially effective in the schedule domain. Because model with HAS was able to recognize argument using various expressions, the number of conversation turns was reduced. These results reflect that the model with HAS does not increase the success rate, but that it is very effective in improving the actual satisfaction.

### 6.2. Comparison with Other Frameworks

As regards the evaluation of the dialog framework, unlike other studies, it is difficult to directly compare the performance of the dialog frameworks. Therefore, we compare the characteristics of each framework. Table 4 shows the comparison of the characteristics of RavenClaw [46], DialogStudio [47], Owlspeak [50], Rasa [51], OpenDial [52], PyDial [53], which are the existing typical dialog frameworks, and the proposed model.

RavenClaw creates a dialog plan by representing the knowledge as a task tree. Then, the dialog engine loads the tasks to be executed onto the dialog stack and calls the routines in sequence. This structure focuses on separating the objective of a dialog into several tasks and managing it in a tree structure. On the other hand, DialogStudio focuses on developing a dialog system based on a corpus because it uses the example-based DM. DialogStudio searches for the most similar example for a given user input. Therefore, it is possible to develop a high-quality dialog system if the example DB built using the tagged corpus is sufficient. On the other hand, if the tagged corpus is insufficient, the quality of the dialog system deteriorates significantly. In addition, it is difficult to predict the results for a particular input because the search results for an input are determined by similarity. Moreover, it is difficult for the developer to precisely control the dialog system; despite the addition of examples that output the desired results, there is no guarantee for them to be selected. Owlspeak is a framework that provides IS-based DM, MVP design pattern, and ontology representation. Owlspeak also employs outstanding development scalability by using universal plug and play (UPNP). IS-based DM provides more generalized domain knowledge and strategy compared to FS-based DM. But FS-based DM with module router functions similarly to IS-based DM. Rasa is a framework focused on developing machine learning-based NLU and machine learning-DM. In particular, Rasa can improve performance at low costs as it requires fewer corpuses for performing initial learning through the application of a bootstrapping technique. In addition, Rasa supports a story graph, which visualizes the path that dialog management can work in the learning process, using a directed graph. This feature can help users identify the characteristics of dialog management. However, Rasa has a similar drawback to Dialog Studio in that it can only use the machine learning method, and it does not support NLG. OpenDial is a framework that can implement the dialog system in an “if ... then ... else ...” approach. OpenDial consists of rules with conditional statements for all the operations of the dialog system and actions with probability; these rules are expressed in XML format. OpenDial provides a function to automatically generate rules from dialog corpus using supervised learning and reinforcement learning. Although it is data-driven, we can interpret the generated model due to this characteristic. On the other hand, OpenDial is difficult to operate effectively given the presence of a large number of rules during the development of a multi-domain dialog system that supports various functions. PyDial is a framework that selectively supports rule-based models and machine learning-based models for each component. In particular, PyDial has the advantage of providing the relatively recently proposed DM, NLU, and NLG. It is a very high-quality framework that can support simulation and evaluation functions.

Because the proposed dialog framework is developed using FS-based DM, it is possible to build a dialog system in a manner that is similar and more intuitive as compared to other frameworks. In addition, because the rule-based and machine-learning-based approaches can be used simultaneously for NLU, it is possible to easily add new tasks without the addition of significant data while providing high performance. Further, the proposed framework provides differentiated functions by using hierarchical arguments and a module router. Hierarchical arguments can contribute to improving the quality of the dialog system; they help the system intelligently respond to various argument representations, that are otherwise difficult to handle using existing dialog frameworks. For situations that necessitate the direct development of a dialog system, existing frameworks must be implemented at the source code level in order to add special functions or perform the desired dialog scenarios, thus rendering the development process more difficult. The module router allows the developer to indirectly control the processing of the dialog system without directly modifying the source code of the dialog framework, thus helping add or manage additional functions more easily. Therefore, this feature considerably reduces the complexity of implementation in our dialog system, resulting in improved productivity.

## 7. Conclusions

In this study, we proposed a dialog framework for developing a task-oriented dialog system. We tested the validity and usability of the proposed framework through evaluations by beginner dialog system developers. The proposed dialog framework provides an environment in which developers without a significant understanding of dialog systems can easily develop a practical dialog system. The rules used in dialogs are expressed as knowledge in an ontological structure, and a basic dialog system can be built using this knowledge. In addition, HAS enables the easy management of various argument expressions, resulting in more intelligent conversations. Moreover, a variety of functions can be added by defining external modules without the need for accessing the source code of the dialog systems. To verify the usability of the proposed dialog framework, we trained developers without prior experience with dialog systems. The developers were tasked with developing task-oriented dialog systems that supported six domains in a smartphone environment. We measured their success quantitatively using the success rate metric, while the user satisfaction was measured using qualitative methods. The experimental results indicate that the proposed framework is effective and easy-to-use.

The proposed dialog framework works quite effectively when developers have implemented knowledge and external modules normally; however, it may be difficult to locate the problematic component if the system is incorrectly implemented. In the future, a dialog framework that is more useful can be expected if it can provide a guide that will help developer-s correct their own errors, or if it can correct errors automatically. In addition, the usability of our editor tools still need to be improved. We are currently developing an editor that supports GUI environment. Once it is complete, a task-oriented dialog system can be developed more effectively.

## Figures and Tables

**Table 1:** Number of dialogs for experiments.

Domain	Dialog	Avg. Sentences
Call	237	2
message	186	2
weather	842	2
navigation	383	3.8
tv_guide	458	2
schedule	438	2

**Table 2:** Evaluation result of NLU.

	Rule-Based	Machine Learning-Based	Hybrid
domain	0.805	0.930	0.924
intent	0.787	0.886	0.878
speech act	-	0.962	0.962
argument	0.838	-	0.838

**Table 3:** Evaluation result of success rate and satisfaction.

Domain	Success Rate without HAS	Success Rate with HAS	Satisfaction without HAS	Satisfaction with HAS
call	0.88	-	3.5	-
message	0.89	-	3.3	-
weather	0.95	0.98	3.3	3.5
navigation	0.90	0.91	3.7	3.9
TV_guide	0.92	-	3.5	-
schedule	0.85	0.88	3.0	3.7
average	0.90	0.91	3.4	3.6

**Table 4:** Comparison of dialog frameworks.

	**RavenClaw [46]**	**DialogStudio [47]**	**OwlSpeak [50]**	**Rasa [51]**
DM	plan	example	information state	machine learning
NLU	rule	machine learning	rule	machine learning
NLG	template	example	template	-
hierarchical argument	no	no	no	no
Development scalability	direct coding	direct coding	universal plug and play	direct coding
	**OpenDial [52]**	**PyDial [53]**	**Proposed Method**	
DM	probabilistic rule	rule & reinforcement learning	finite state	
NLU	probabilistic rule	rule & machine learning	hybrid of rule & machine learning	
NLG	probabilistic rule	rule & machine learning	template	
hierarchical argument	no	no	yes	
Development scalability	direct coding	direct coding	indirect coding (with module router)	
